# Listen to PaO_2_/FiO_2 _ratios: they tell us about length of stay

**DOI:** 10.1186/cc10696

**Published:** 2012-03-20

**Authors:** V Inal, B Comert, L Yamanel

**Affiliations:** 1 GATA, Ankara, Turkey

## Introduction

Classification of respiratory distress has been dependent on PaO_2_/FiO_2_; that is, <300 acute lung injury (ALI) and <200 acute respiratory distress syndrome (ARDS). In this study, PaO_2_/FiO_2 _was analyzed for predicting ICU patients' length of stay (LOS).

## Methods

Data of 273 patients admitted to the ICU with RI were retrospectively analyzed for LOS in the ICU. Patients admitted to the emergency department (ED) with RI, documented arterial blood gas analysis (ABGA), and hospitalized in the ICU were eligible for this study within 4 years. The first ABGA in ED PaO_2_/FiO_2 _were taken for predicting ICU LOS. Patients' comorbid diseases, APACHE II/Glasgow scores, non/invasive mechanical ventilation supports were not included in the analysis. Patients were classified into three groups as: (1) >300 not having RI, (2) <300 ALI, (3) <200 ARDS; they were then compared for predicting ICU LOS, and also receiver operating curve (ROC) analysis and area under curve (AUC) were calculated.

## Results

Analysis showed statistical significance of *P *< 0.01 for all groups pointing out that ED ABGA PaO_2_/FiO_2 _levels negatively affected patients' LOS in the ICU. ROC analysis of PaO_2_/FiO_2 _for LOS showed significant AUC: 0.917 levels, which was predicted as a powerful indicator. Patients' data are presented in Table 1.

**Table 1:** Patient data

(*n*)	Age (years)	>300 (*n*) LOS (days)	ALI (*n*) LOS (days)	ARDS (*n*) LOS (days)
Male 165	65 ± 8.2	47 8 ± 2.1	65 12 ± 3.4	53 16 ± 4.2
Female 108	69 ± 7.6	33 7 ± 2.9	43 11 ± 3.7	32 17 ± 3.8

## Conclusion

We concluded that the PaO_2_/FiO_2 _ratio was a powerful indicator for predicting ICU LOS in patients with RI. In addition there was no need to classify patients according to PaO_2_/FiO_2 _to predict LOS; any decreased ratio meant a longer LOS. However, this study was weak in power; it had a small sample, did not include comorbid conditions, did not account for accepted scoring systems, and did not include daily ABGA for prediction. On the other hand, these results are promising for future observations that ABGA taken in the ED would be a supplemental tool for the physician's approach in the ICU.

